# A paired training curriculum and internal monitoring program for clinical research regulatory compliance in the emerging era of the single Institutional Review Board

**DOI:** 10.1017/cts.2017.12

**Published:** 2017-10-09

**Authors:** T. Che Jarrell, Frances Hamblin, Daniel E. Ford, Sylvia R. Powell, Jonathan M. Ellen, Neil A. Goldenberg

**Affiliations:** 1 Clinical and Translational Research Organization, Johns Hopkins All Children’s Hospital, St. Petersburg, FL, USA; 2 Department of Medicine, Johns Hopkins University School of Medicine, Baltimore, MD, USA; 3 Johns Hopkins All Children’s Hospital, St. Petersburg, FL, USA; 4 Department of Pediatrics, Johns Hopkins University School of Medicine, Baltimore, MD, USA

**Keywords:** Clinical research, training, compliance, monitoring

## Abstract

**Background:**

Academic health systems and their investigators are challenged to systematically assure clinical research regulatory compliance. This challenge is heightened in the emerging era of centralized single Institutional Review Boards for multicenter studies, which rely on monitoring programs at each participating site.

**Objective:**

To describe the development, implementation, and outcome measurement of an institution-wide paired training curriculum and internal monitoring program for clinical research regulatory compliance.

**Methods:**

Standard operating procedures (SOPs) were developed to facilitate investigator and research professional adherence to institutional policies, federal guidelines, and international standards. An SOP training curriculum was developed and implemented institution-wide. An internal monitoring program was launched, utilizing risk-based monitoring plans of pre-specified frequency and intensity, assessed upon Institutional Review Boards approval of each prospective study. Monitoring plans were executed according to an additional SOP on internal monitoring, with monitoring findings captured in a REDCap database.

**Results:**

We observed few major violations across 3 key domains of clinical research conduct and demonstrated a meaningful decrease in the rates of nonmajor violations in each, over the course of 2 years.

**Conclusion:**

The paired training curriculum and monitoring program is a successful institution-wide clinical research regulatory compliance model that will continue to be refined.

## Introduction

The conduct of clinical research at academic health centers offers great potential benefit with regard to advancing knowledge, improving future patient outcomes, developing and advancing the careers of faculty, trainees, and research professionals, and enhancing institutional reputation. However, with these great potential benefits come significant potential risks—to participants (e.g., patients), investigators, and institutions. It is an ethical responsibility of the clinical investigator and the institution that engages in clinical investigation to adopt a comprehensive approach toward safeguarding participants in clinical research, and assuring quality of the research process.

Presently, for-cause and random audits are the norm for clinical research regulatory compliance monitoring. At academic centers, these audits are typically performed by the institution’s Office of Human Subjects Research or equivalent (ie, the “IRB office”) as well as (e.g., in cancer studies) by a Cancer Center, where one exists. As a rule, audit findings are not shared beyond the institution, apart from any applicable reportability requirements to the study sponsor, the United States Food and Drug Administration (FDA), and/or the federal Office of Human Research Protections. By contrast, FDA publically posts on its Web site Warning Letters addressed to individual investigators, as well as each Form 483s (Notice of Inspectional Observations), stemming from its individual study audits [1, 2]. However, aggregate data on change in frequency and severity of violations over time have not been published for the current paradigm of random and for-cause audits. The success of such an approach should, therefore, be questioned, and an alternative approach of routine, systematic internal monitoring is worthy of evaluation. Such an alternative approach is particularly timely given the recent National Institutes of Health (NIH) policy on centralized “single Institutional Review Boards” (sIRBs) for multicenter studies [3]. The desired benefit of the sIRB model is greater efficiency and consistency in multicenter study implementation; however, the diminution of local IRBs’ oversight role for site-specific study conduct requires that the local institution has in place (or develops) an adequate monitoring program for site participation in such studies.

In this communication, our objective is to describe the development, implementation, and outcome measurement of an institution-wide paired training curriculum and internal monitoring program for clinical research regulatory compliance, as a major component of one pediatric academic health center’s solution to the challenge of reducing systematic risks to clinical research participants, investigators, and institutions. However, we propose this solution as a model for potential adoption by other academic health centers. Although the training curriculum and monitoring program leverage an established, robust, centralized institutional infrastructure model (a multiunit Clinical and Translational Research Organization [CTRO] described previously [4]) designed to support investigators in the conduct of both investigator-initiated and pharmaceutical industry-driven clinical research studies, a CTRO or similar centralized infrastructure for clinical research execution is neither necessary nor sufficient for the training curriculum and monitoring program to fulfill the mandate for enhanced clinical research oversight.

## Methods

### Setting

Johns Hopkins All Children’s (JHAC) Hospital is a pediatric academic health system whose center is located in St. Petersburg, FL, and home to one of three stand-alone Children’s Hospitals in the state of Florida. The health system features a 259-bed tertiary care hospital; an adjacent Outpatient Care Center with over 200,000 visits annually; an adjacent, 225,000 square-foot Research and Education facility under construction (to be completed in 2018); multiple clinical outreach centers providing nearly 150,000 additional visits per year; and several critical and noncritical care units within hospitals in the Tampa Bay area and beyond. Overall, JHAC health system serves a catchment area of 17 counties and a population of 1.3 million children.

The health system integrated into Johns Hopkins Medicine in 2011, marked by: the recruitment of full-time Hopkins faculty to the JHAC campus beginning in early 2012; the integration of the IRB into the Johns Hopkins Medicine IRB system in 2013; and the establishment in 2014 of 4 initial institutes (Cancer and Blood Disorders Institute, Heart Institute, Institute for Brain Protection Sciences, and Maternal Fetal and Neonatal Institute) and 3 departments (Medicine, Surgery, and Anesthesia). The institute model encompasses all mission legs (research, education, clinical excellence, and advocacy) and numerous disciplines and subspecialties collaborating in the execution of those missions (e.g., cardiology, cardiothoracic surgery, cardiac anesthesiology, cardiac intensive care, etc. in the Heart Institute). The departments function as the primary administrative and academic homes for faculty who were not primarily aligned with one of the institutes. From 2013–2014, concomitant with the establishment of the JHAC CTRO and its component units, the paired training curriculum and internal monitoring program for clinical research regulatory compliance were developed within the Research Regulatory Affairs and Quality Assurance Unit, in close collaboration with the Research Operations Unit and Investigational Drug Services Unit of the CTRO. Since 2014, JHAC has averaged ~220 active prospective studies across a total of ~60 principal investigators (PIs) in child health and disease at any given time, and has devoted an institutionally-supported total of 1.2 full-time equivalency (FTE) toward the ongoing implementation of the training curriculum and the internal monitoring effort around these studies.

### Development and Implementation of Standard Operating Procedures (SOPs) and Associated Training Curriculum

The first step in the development of the training curriculum and monitoring program was the development and implementation of a cadre of SOPs (topics listed in Table 1) that are institutionally tailored to facilitate investigator, trainee, and clinical research professional adherence to institutional policies, federal guidelines, and international standards in clinical research. Next, we developed 3 curricular components for SOP training, each of which included both didactic and interactive components: Regulatory Affairs/Quality Assurance Rounds (bi-monthly); “Brown-Bag” Training Series on SOPs (monthly); and a Training Mini-Retreat on Investigator Responsibilities in FDA-Regulated Drug and Device Trials (annually, by institute and department). The first component was initiated in 2014, the second in 2015, and the third in early 2016.

**Table 1:** Standard operating procedures (SOPs)

SOP ID	Title	Purpose
RegQA001	Management of Clinical Trial Regulatory Documentation	Describe standardized procedures for regulatory file management, including sponsor-investigator regulatory files (when applicable)
RegQA002	Oversight on Conduct of External Audits/Monitoring Visits (By Sponsors or Other External Monitors/Auditors)	Define a consistent and uniform framework for preparing and hosting an external audit
RegQA003	Investigational Drugs and Devices Auditing/Monitoring	Define a consistent and uniform framework for investigational product accountability monitoring of the institution’s Investigational Drug Services Unit
RegQA004	Informed Consent Discussion and Documentation	Establish a standardized method for executing and documenting the informed consent process consistent with applicable regulations, policies, and guidelines
RegQA005	Triage of an Acute Health Concern in a Research Setting	Establish a framework for clinic staff to communicate to the appropriate research study staff any acute health concern occurring on a patient or participant who is simultaneously participating in a clinical study
RegQA006	Investigational Medical Device Chain of Custody and Related Documentation	Describe procedures for ordering, receiving, storing, dispensing, use, returning, shipping, and disposing of investigational medical devices in clinical trials
RegQA007	Training for Study Coordinators, Clinical Unit Based Research Nurses (CUBRN), Principal Investigators and Other Designated Clinical Research Personnel	Define research-related training requirements for all new and currently employed research personnel
RegQA008	Submission of Non-Adverse Event (AE) Safety Documentation to the Institutional Review Board (IRB)	Describe procedures for review and reporting of non-adverse event safety documentation to the IRB
RegQA009	AE Surveillance and Reporting to the IRB	Describe procedures for surveillance, determination, and reporting of submission of AEs to the IRB
RegQA010	Principal Investigator (PI) and Site Investigator Responsibilities for the Conduct of Human Subjects Research Conducted at More than One Johns Hopkins Medicine (JHM)/JHM-Affiliated Site Under the Direction of One JHM PI	Describe procedures for PI and participating site lead investigator responsibilities and communication when more than one JHM participating sites are involved in one JHM IRB-approved application
RegQA011	Research Note to File	Provide a good documentation practice (GDP)-based method for drafting study-related notes-to-file
RegQA012	Exception and Deviation Reporting	Describe procedures for facilitating adherence to protocol deviation and exception reporting to the IRB and study sponsor
RegQA013	Routine Internal Monitoring of IRB-Approved Prospective Clinical Research Studies	Describe procedures for routine internal monitoring of IRB-approved prospective clinical research studies by personnel in the Regulatory Affairs or Quality Assurance Unit of the Johns Hopkins All Children’s (JHAC) Clinical and Translational Research Organization
RegQA014	Development, Implementation, Monitoring, and Closure of Corrective and Preventative Action (CAPA) Plans	Describe procedures for assessing root cause(s) of, and prevention strategies for, substantive study-specific problems through voluntary or IRB-required CAPAs
RegQA015	Research Chart Organization and Case Report Form Completion	Describe standardized procedures for organizing and compiling study participant research charts and for completing case report forms
RegQA016	Documentation of Eligibility Criteria Utilizing an Eligibility Checklist	Describe standardized procedures for verifying eligibility criteria utilizing an Eligibility Checklist
RegQA017	CTEP Investigator Registration and Renewal Process	Describe procedures for NCI Cancer Therapy Evaluation Program (CTEP) investigator registration renewal for JHAC-based PIs
RegQA018	Clinical Trial Registration and Reporting on www.ClinicalTrials.gov	Describe clinicaltrials.gov registration procedures for IRB-approved clinical trials led by JHAC-based PIs

An internal monitoring program, codified in an additional SOP and associated tools, was designed and launched at the end of 2014. The internal monitoring procedure begins with the assessment of monitoring category (Table 2) and associated monitoring frequency and scope/intensity for a given study. This assessment is performed at the time of IRB approval, and is largely based on the IRB’s risk categorization of the study. A corresponding Monitoring Plan is then drafted for the study, using a template provided as an appendix to the SOP on Internal Monitoring. The study’s PI and the Chief Research Officer both review and sign-off on the Plan, with copies provided to the primary clinical research coordinator (CRC) as well as to the regulatory research assistant, to whom responsibility is designated for filing the plan in the study’s Regulatory Binder. For studies conducted under an Investigational New Drug or Investigational Device Exemption, an additional monitoring visit is conducted by the auditing/monitoring staff of the Office of Human Subjects Research (“IRB office”), or via the CTRO-based internal monitoring program in the case of a trial that utilizes an external sIRB.

**Table 2:** Assigned level, frequency and intensity of internal monitoring, by study type

Routine internal monitoring level	Prospective study type(s)	Routine internal monitoring frequency/intensity
1	Observational study Interventional study not conducted under an Investigational New Drug (IND)/Investigational Device Exemption (IDE)	Approximately every 18–24 months Eligibility/consent review of 10–25% of study participants
2	Interventional study conducted under an IND/IDE, in which the IND/IDE sponsor is not a Johns Hopkins All Children’s (JHAC) Hospital-based Investigator Non-high-risk study approved by the National Cancer Institute (NCI) Pediatric Central Institutional Review Board (PedCIRB)	Approximately every 12–18 months Eligibility/consent review of 100% of study participants Primary efficacy or safety endpoint review of 50–75% of study participants
3	Interventional study conducted under an IND/IDE, in which the IND/IDE sponsor is a JHAC-based investigator* High-risk study approved by the NCI PedCIRB	Approximately every 6–12 months Eligibility or consent review of 100% of study participants Primary efficacy or safety endpoint review of 75–100% of study participants

* A study set-up review is also conducted by Office of Human Subjects Research, before the study is open for enrollment.

Approximately three months before the first, and each subsequent monitoring episode, the PI and CRC are notified via email that a monitoring episode is planned, and a period of one to two days (depending on pre-specified scope) is collaboratively scheduled in which the PI and/or CRC will be available to address any questions from the monitor during the monitoring process. Following completion of the monitoring episode and its write-up (using a standardized documentation tool provided as an Appendix to the SOP on Internal Monitoring), a wrap-up meeting is conducted with the PI, CRC, monitor, Director of Research Operations, and the Chief Research Officer, to review the observations and provide any necessary focused re-training for the PI and CRC on SOP components that relate to the nature of violations observed. Any violations that meet requirements for reportability to the IRB are then duly reported to the IRB by the PI with support from the CRC and regulatory research assistant.

### Database Design, Data Collection, and Outcomes Analysis

In late 2014, just before the launch of the monitoring program, a database was designed on a web-based electronic data-capture system (REDCap) and implemented for capture of discrete data on the observations from each monitoring episode, for each study monitored. Data collection included (but was not limited to) the following: IRB approval number, investigator name, institute or department primary affiliation, therapeutic area, study monitoring category (see Development and Implementation of Standard Operating Procedures (SOPs) and Associated Training Curriculum section and Table 2), and numerator and denominator data for each domain monitored (number of research participants monitored for a given domain, number of monitored participants for whom violations were found for that domain, respectively). Violations were further categorized as “major” Versus “nonmajor,” as shown in Table 3, modeled from the National Cancer Institute Cancer Therapy Evaluation Program criteria [5]. The 3 pre-specified domains for initial program evaluation were eligibility enforcement or informed consent process documentation; adverse event determination, documentation, and reporting; and investigational drug procedural and environmental controls and accountability. Statistical analyses compared frequencies of violations for a given domain between calendar years 2015 and 2016, using Yates’ χ^2^ testing, corrected for continuity; in the case of cell values <5 in 2×2 tables, Fisher’s exact test was instead employed. A *p*-value of <0.05 was established as the a priori threshold for statistical significance (ie, α level).

**Table 3:** Examples of major and nonmajor violations by domain

Category	Major violations	Nonmajor violations
Eligibility and informed consent	An enrolled study participant did not meet all eligibility criteria within protocol-specified timeframe (or authorization for protocol exception not obtained from sponsor and Institutional Review Board (IRB) before enrollment) Signed informed consent document not present in study participant’s research chart, or lacks signature or date of signature before enrollment by a study participant or legally authorized representative or the appropriately authorized study team member who obtained informed consent Informed consent not obtained in a language comprehended by a study participant or legally authorized representative (or an appropriate IRB-approved translated form was not used) Informed consent document used was not the current IRB-approved version Re-consent of study participant was not obtained in a circumstance and/or timeframe required by the IRB	Eligibility source documentation missing on an enrolled study participant Informed consent document contains signatures and dates of signatures before enrollment from study participant or legally authorized representative and appropriately authorized study team member, but has errors in placement of signatures or has missing initials or dates Consent form does not bear a unique subject identifier label Signed informed consent document not scanned into the electronic health record, if the study participant is a patient of the health system
Adverse event and other safety reporting	Protocol-specified baseline assessment (including testing) relevant to subsequent study participant adverse event (AE) monitoring not performed Follow-up assessments (including testing) relevant to study participant AE monitoring not performed Grades, types, or dates or duration of serious AEs* not accurately documented or determined, missing substantive source documentation, or not reported within prescribed timelines to IRB and sponsor [and to FDA, in the case of a sponsor-investigator role under an Investigational New Drug (IND)/Investigational Device Exemption (IDE)] Unanticipated problems involving research participants or others not reported within prescribed timelines to IRB and sponsor Systemic under-reporting or incomplete/inaccurate determination/reporting of nonserious AEs*, including protocol-specified laboratory results that impact study participant safety monitoring Repetitive failures or substantive delays in submitting relevant safety reports (including Data and Safety Monitoring Board reports, as applicable) to the IRB (and to FDA and investigators at participating sites, as applicable, in the case of a Sponsor-Investigator role under an IND/IDE).	Unreported or incompletely or inaccurately determined or reported nonserious AEs, unless judged to be systemic in nature
Investigational drug/device	Study intervention (drug/device regimen) or related protocol-specified treatments not administered according to study protocol, with regard to correct drug/device, dose, or route of administration, or not documented Systemic incompleteness or inaccuracies in documentation of study intervention and/or related protocol-specified treatments Study intervention (drug or device regimen) not ordered by appropriately credentialed clinician member of the study team Inadequately justified protocol deviation with regard to timing of study intervention or related protocol-specified treatments Inadequately justified protocol deviation with regard to prohibited medications Systemic incompleteness or inaccuracies in documentation of chain of custody of investigational drug/device (includes documentation of initial inventory relative to shipping log, if supply received from sponsor/sponsor designee; also includes documentation of destruction of investigational drug/device or its return to sponsor/sponsor designee) Inadequately justified protocol deviation with regard to return/destruction of unused investigational product to Sponsor/Sponsor designee.	Study intervention and related treatment documented, but documentation is incomplete—unless judged to be systemic in nature Protocol deviation involving timing of study intervention or related protocol-specified treatments justified, but not documented/reported Protocol deviation involving prohibited medications justified, but not documented/reported Incomplete or inaccurate documentation of chain of custody of investigational drug or device (includes documentation of initial inventory relative to shipping log, if supply received from sponsor or sponsor designee; also includes documentation of destruction or return to sponsor or sponsor designee—unless judged to be systemic in nature Protocol deviation involving return/destruction of unused investigational product to sponsor/sponsor designee justified, but not documented/reported

* If the IRB-approved protocol specifies that a given AE is not reportable, then lack of documentation, determination, or reporting of that AE does not constitute a violation.

## Results

**Fig. 1:**
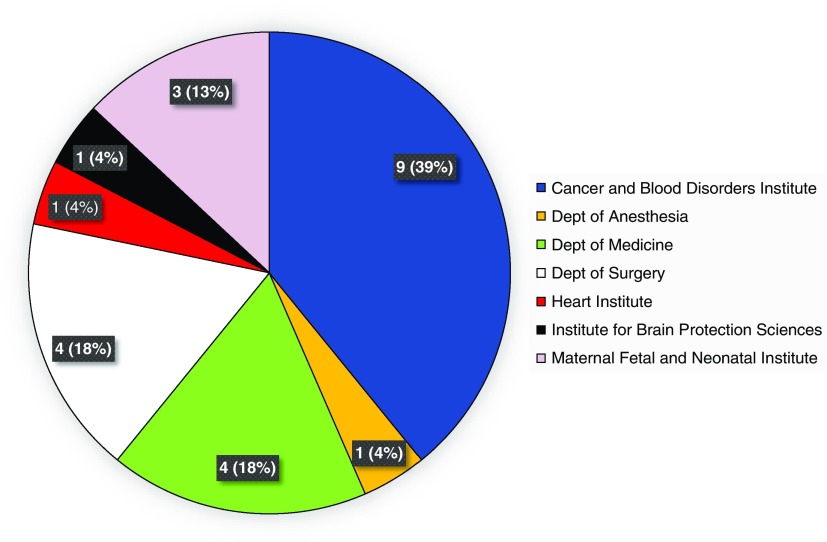
Unique studies monitored during calendar years 2015 and 2016, by institute and department.

During the evaluation period of calendar years 2015 and 2016, 23 unique studies were internally monitored via the program, across 13 therapeutic areas. This represented the number of prospective studies that had undergone de novo IRB approval or approval of a change-in-research submission, and had reached the pre-specified interval(s) for monitoring. Fig. 1 provides a breakdown of studies monitored, by institute and department. Therapeutic areas represented by these studies included the following: bone marrow transplantation, n=1; hemostasis and thrombosis, n=2; hematological malignancies, n=3; hernia repair, n=1; central nervous system (CNS) tumors, n=2; stroke, n=1; neonatology, n=3; appendicitis or cholecystitis, n=2; chest wall deformity, n=2; congenital or acquired heart disease, n=1; immunodeficiency, n=3; infectious disease, n=1; and multidisease studies, n=1.

**Fig. 2:**
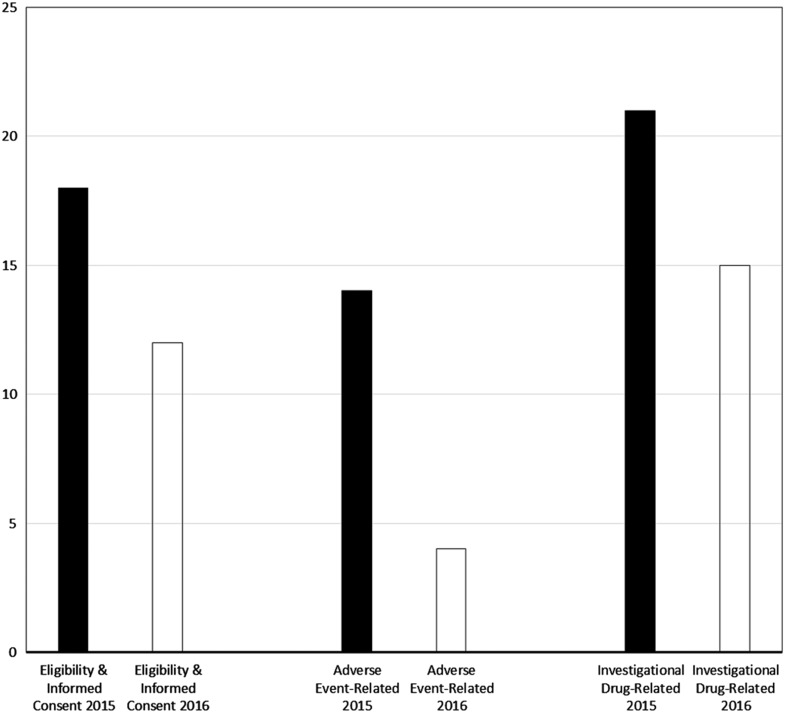
Proportion of monitored study participants for whom violations were found, by domain and calendar year of program evaluation. Note: nearly all violations were nonmajor (see also Methods section and Table 3).

Major violations were rare, at 0.3% (1/407) for eligibility enforcement or informed consent process documentation, 0.5% (1/191) for adverse event determination, documentation, and reporting, and at 0% (0/42) for investigational drug procedural and environmental controls and accountability. As shown by the bar graphs of Fig. 2, the percentage of monitored participants for whom eligibility or informed consent violations were identified (predominantly minor, given the aforementioned rarity of major violations) declined from 18% (20/112) in 2015 to 12% (34/295) in 2016 (*p*=0.13). Similarly, the proportion of monitored participants for whom adverse event violations were disclosed decreased over the 2-year period, from 14% (8/56) to 4% (6/135); this decrease was statistically significant (*p*=0.04). Finally, the frequency of investigational drug-related violations was reduced from 21% (6/29) in 2015 to 15% (2/13) in 2016 (*p*=1.0).

## Discussion

In this report, we have described the development, implementation, and outcome measurement of an innovative, institution-wide, paired training curriculum, and internal monitoring program for clinical research regulatory compliance, as a major component of one pediatric academic health center’s solution to the challenge of reducing systematic risks to clinical research participants, investigators, and institutions. The paired training curriculum and monitoring program reflect the institution’s continual efforts to prioritize and optimize patient safety. We have measured a very low rate of major findings postimplementation, and demonstrated a meaningful decrease—over a short period of two years of monitoring—in the rates of nonmajor violations across each of three key domains of clinical research conduct: eligibility criteria enforcement and informed consent process documentation; adverse event determination, documentation, and reporting; and investigational drug procedural and environmental controls and accountability among clinical trials that involve investigational drugs. Given the relatively small number of monitoring episodes conducted to date, statistical significance was demonstrated only for the decline in adverse event violations, despite the substantive relative reductions in violations across all domains evaluated. Nevertheless, encouraged by these results, we are continuing to support this initiative, and as of mid-2017 we are implementing an additional monitoring visit systematically after the enrollment of the first patient in all category-2 and -3 studies. This refinement to the monitoring program, we believe, will reduce the number of minor violations identified to date that pertain to missing or incomplete standardized documents specified in our SOPs, such as eligibility checklists and master adverse event logs. As further data accrue from the monitoring program, we will seek to identify systemic trends that yield opportunities for additional refinements to the monitoring program, targeted institution-wide re-training on corresponding SOPs, and further optimization of SOPs, as warranted.

We believe that the training curriculum and monitoring program are scalable, in which our example of 1.2 FTE devoted to a program that involves on average 220 prospective studies among 60 investigators, could be increased or decreased commensurately with the size of the clinical research faculty and prospective study portfolio of a given academic health center at a given time. Although a CTRO or similar centralized infrastructure for clinical research execution is neither necessary nor sufficient for the training curriculum and monitoring program to fulfill the mandate for enhanced clinical research oversight, it is our opinion (informed, in part, by experience) that the presence of a CTRO is a key facilitator of the achievement of metrics of success, such as those reported here. It is our aim to continue to monitor outcomes of the curriculum and program, and to continually refine the program as needed in order to optimize the metrics of success.
